# When conventional approach in toxicity assays falls short for nanomedicines: a case study with nanoemulsions

**DOI:** 10.1007/s13346-024-01776-7

**Published:** 2025-01-08

**Authors:** Ines Nikolić, Jelena Đoković, Dora Mehn, Giuditta Guerrini, Snežana Savić, Olivier Jordan, Gerrit Borchard

**Affiliations:** 1 Faculty of Science, School of Pharmaceutical Sciences, University of Geneva, Geneva, Switzerland; 2 Faculty of Pharmacy, Department of Pharmaceutical Technology and Cosmetology, University of Belgrade, Belgrade, Serbia; 3 European Commission, Joint Research Centre (JRC), Ispra, Italy

**Keywords:** Nanomedicines, Nanoemulsions, PEGylation, In vitro cytotoxicity, Orthogonal techniques, Critical quality attributes

## Abstract

The aim of this study was to assess the critical quality attributes of parenteral nanoemulsion formulations by measuring several physicochemical parameters and linking them to their in vitro performance, illustrating how simplistic and routinely used approaches are insufficient for understanding a potential nanomedicine. Physicochemical characterization should encompass size and size distribution through at least two orthogonal techniques, such as dynamic light scattering (DLS) and electron microscopy, with added value from analytical ultracentrifugation. In vitro toxicity assessment was performed using three different assays to determine mitochondrial activity (WST-1), membrane integrity (lactate dehydrogenase release (LDH) assay), and cell viability (propidium iodide (PI) staining). Special focus was placed on estimating appropriate incubation times for relevant results in biological investigations. All formulations had an average diameter of around 100 nm. Conclusions regarding in vitro safety were assay-dependent: LDH and PI-based assays showed good correlation, while the WST-1 assay indicated that the non-PEGylated formulation altered mitochondrial activity more significantly compared to the PEGylated ones. The study underlined that the selection of appropriate cytotoxicity assays should be based on the possible mechanism of cellular perturbation. Alternatively, different aspects of cellular toxicity should be tested. Additionally, there is a need for well-designed controls to overcome nanoparticle scattering effects and avoid potentially false high toxicity results, which was demonstrated. Combining orthogonal, well-designed physicochemical and biological assays in a standardized manner as an initial step in the reliable preclinical characterization of nanomedicines is suggested. This represents a key aspect of new methodologies in nanomedicine characterization.

**Graphical abstract:**

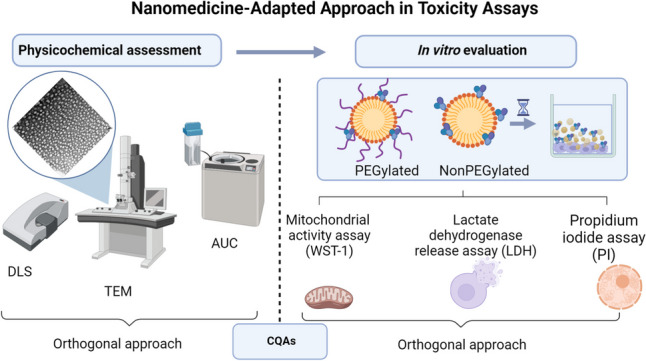

**Supplementary Information:**

The online version contains supplementary material available at 10.1007/s13346-024-01776-7.

## Introduction

In vitro biological evaluation is an important aspect of nanomedicine characterization, and the need for reliable methodologies suitable for the early assessment of nanomedicine candidates is a *condition sine qua non* for their smoother translation towards the clinics. In vitro tests are considered essential tools for toxicity screening, both in basic research and for regulatory purposes [1]. Recent surveys conducted among the regulatory authorities have confirmed that this area is of high importance, encompassing diverse biological aspects such as hemotoxicity, immunogenicity, complement activation, and inflammation [2].

Accordingly, for better communicating the type of information considered relevant for regulatory decision-making, the Committee for Medicinal Products for Human Use (CHMP) of the European Medicines Agency (EMA) published reflection papers addressing several different nanomedicinal products: intravenous liposomal products [3], block copolymer micelle products [4], intravenous iron-based nano-colloidal products [5], as well as the reflection paper addressing the coating of nanomedicines [6]. A similar approach has been taken by the US Food and Drug Administration (FDA) in the guidance related to liposomal drug products [7] and the guidance related to drug products containing nanomaterials [8]. Within these documents, critical quality attributes (CQAs) of these specific nanomedicinal products and their relevance for product evaluation have been suggested.

In this context, in vitro cytotoxicity assessment has been recognized as one of the first and main tasks in preclinical safety and efficacy characterization of nanomedicines or nano-enabled medicinal products. It is primarily performed through in vitro cell-based toxicity studies, requiring robust, predictive, and reliable methods [9]. Even though regulators have defined their requirements, there is still a lack of methodologies that may be used to assess a nanomedicine candidate. Some promising methods/protocols have been developed by relevant organizations and used by product developers [10, 11], though they have not yet been established as the binding practice by the regulators [9, 12]. For example, there is no regulatory document that directly addresses in vitro toxicity testing specifically for nanomedicines. Reflection papers issued by the EMA [3, 5] emphasize the importance of in vitro toxicity testing as a part of the safety assessment for nanomedicines to identify potential risks before entering the in vivo investigation, but do not specify exact assays or criteria.

Even though currently available protocols recommended for nanomedicine safety assessment consider renal and hepatic cell lines as suitable platforms, representing the main elimination organs, the probability of detecting immunotoxicity of nanomedicines increases as they move from the preclinical to the clinical phases of investigation [13]. Therefore, having an appropriate surrogate for detecting immunotoxicity is of high importance. Additionally, it has been suggested that valuable pieces of information may be obtained by assessing standard toxicity assays employing macrophages [11].

Furthermore, nanomedicines possess properties that could affect the results of certain biological assays used to assess their toxicity. In this context, interactions between the test reagents and the nanomaterial have been identified as one of the most important issues in toxicity testing that influence the market authorization of nanomedicines [14]. For example, interactions of doxorubicin-loaded liposome formulations in colorimetric cytotoxicity assessment have been reported. Consequently, it is not possible to detect dose-response curves, and, as a result, false negative results are provided. And this is just one example next to similar observations by other authors [15, 16]. These physicochemical interactions of the nanostructures with the assay readouts are especially highlighted in colorimetric assays, which are usually the first steps in (nano)toxicity assessment. Accordingly, in its documents related to the nonclinical studies of drug products containing nanomaterials, FDA indicates that some routinely used in vitro assays may not be appropriate and may require adjustments [8].

Another important aspect that needs to be considered is that the biomolecular identity of the nanoparticles can be significantly altered once they enter the biological/biorelevant environment. Due to the biomolecules present in the cell culture media, as well as those secreted by the cells, the size and surface aspects of the nanoparticles can be changed, influencing the interaction with the cells and/or leading to particle aggregation. This new (“biological”) identity of the particle is usually very different from the inherent (“chemical” or “synthetic”) identity and more challenging to be analyzed [17]. Moreover, in most in vitro studies, it is considered that the initial concentration of the nanoparticles deposited on the cells is the effective one. However, since nanoparticles are not simple molecules that are dissolved in the cell media, and their transport to the cell monolayer is highly dependent on their physical properties as well as on those of the surrounding medium, the number of nanoparticles that effectively reach the cells may differ from the nominal amount. Therefore, dosing within in vitro experiments is crucial for obtaining highly relevant results since it is directly linked to cellular uptake [1].

In the presented work, the authors aimed to go through different approaches in cytotoxicity evaluation of a defined set of nanoformulations (parenteral nanoemulsions prepared *via* high-pressure homogenization process), comparing and adjusting the available protocols, or establishing new ones, which may be considered more suitable for nanomedicines. Motivated by the fact that nanomedicines have a complex behaviour, a special experimental segment was devoted to the evaluation of the exact fraction of the sample that is reaching the cell monolayer. Finally, the best combination of assays and protocol designs are presented aiming to provide fast and reliable conclusions on nanomedicine toxicity (relying on this case study) in a cost-effective manner. Having in mind the latest strategies underlying the inclusion of immune system cell lines for toxicity assessment, the one selected for the experiments presented here was RAW 264.7 (murine macrophage cell line).

Given the continuous development of the nanomedicine market [18], it is not surprising that it includes various carriers and formulation types, such as:

i) lipid-based carriers (e.g., liposomes, multiple generations of lipid-based nanoparticles - including the recent Onpattro^®^ and COVID-19 vaccine formulations, nanoemulsions for drug delivery or parenteral nutrition) [19–21],
ii) polymer-based drugs [22],
iii) drug nanosuspensions (nanocrystals and amorphous dispersions) [23–25].
iv) protein-bound drugs [26], and.
v) lipid-polymer hybrid nanoparticles - as the latest generation [27].

These systems encompass numerous administration routes and indications - predominantly in oncology and immunology [28]. Nano-enabled medicinal products face different challenges – both in research and in the regulatory framework [29–32]. Therefore, the authors aim to provide a contribution to the field of these innovative products for their smoother translation to the clinical application. In our study, only blank formulations (without the active pharmaceutical ingredient, API) were tested. The rationale behind this approach lies in the unique physicochemical characteristics of nanostructured carriers, which need to be evaluated independently of the API. Regulatory frameworks emphasize a “quality-by-design” approach, which requires rigorous safety assessments of both drug-loaded and placebo formulations to differentiate between the toxicity effects of the nanomaterial itself and those related to the API [33–36]. By focusing on placebo formulations, our study establishes a solid understanding of the carrier’s properties, which can then be applied to API-loaded formulations using the same principles. This step-wise approach ensures that the potential toxicity of the carrier is fully understood before progressing to more complex formulations, thereby supporting the development of safer and more effective nanomedicines.

## Methodology

### Preparation of nanoemulsions

Nanoemulsions (Table 1) were prepared via high pressure homogenization, as previously described by Đoković et al. [37]. Briefly, the oil phase (consisting of medium chain triglycerides (MCT), soybean oil, soybean lecithin and butylhydroxytoluene) and the aqueous phase (composed of polysorbate 80, glycerol, sodium oleate, and highly purified water) were separately prepared and heated to 50 ˚C. The aqueous phase was then poured into the oil phase, pre-homogenized at 11,000 rpm for 1 min applying a rotor-stator homogenizer (IKA^®^-Werke GmbH & Co. KG, Staufen, Germany) and then further homogenized using a high-pressure homogenizer (EmulsiFlex-C3, Avestin Inc., Ottawa, Canada) at 800 bars for 10 cycles. PEGylated formulations were prepared in the same manner, just the PEGylated phospholipids were added either to the oil phase (PEG2000-DSPE) or to the aqueous phase (PEG5000-DPPE). After preparation, nanoemulsions were packed in 20 ml glass vials, and sealed.

**Table 1:** Qualitative and quantitative composition of the nanoemulsion formulation

	Nanoemulsion without PEG layer	PEGylated nanoemulsion	
**Ingredients (**%, *w/w***)**	**NonPEG**	**P21**	**P51**
Soybean oil	4	4	4
Medium chain triglycerides	16	16	16
Soybean lecithin	2	2	2
Butyl hydroxytoluene	0.05	0.05	0.05
PEG2000-DSPE^*^	-	0.1	-
PEG5000-DPPE^**^	-	-	0.1
Polysorbate 80	2	2	2
Glycerol	2.25	2.25	2.25
Sodium oleate	0.03	0.03	0.03
Ultrapurified water	to 100	to 100	to 100

NonPEG, P21 and P51 are the designations for the three formulations examined and whose compositions are detailed in Table 1

*N−(carbonyl−methoxypolyethylene glycol−2000)−1,2−distearoyl−sn−glycero−3−phosphoethanolamine, sodium salt

**N−(carbonyl−methoxypolyethylene glycol−5000)−1,2−dipalmitoyl−sn−glycero−3−phosphoethanolamine, sodium salt

### Physicochemical characterization

Nanoemulsion formulations were subjected to the set of physicochemical characterization techniques to obtain insight into the difference between PEGylated and nonPEGylated formulations, as well as to help interpretation of the results of in vitro toxicity experiments.

#### Dynamic light scattering (DLS)

For the initial sizing experiments, DLS characterization technique was performed, applying Zetasizer Nano ZS (Malvern Instruments, UK), relying on the DLS EU-NCL protocol [38]. Prior to the measurement, all samples were diluted in the MilliQ water (filtered through a 0.22 μm filter before being mixed with the sample), so that the attenuation and detector count rate were at appropriate levels, with intercept of the correlation function higher than 0.95. For all samples, optimal dilution was at the ratio 1:500 (*v/v*). Measurements were conducted at 25 °C, laser wavelength was 633 nm, applying a back scattering detector (173 °). Equilibration time was set to 5 min. Results were presented as Z-average (Z-ave) with polydispersity index (*n* = 10) ± standard deviation.

#### Nanoparticle tracking analysis (NTA)

NTA was selected as complementary to the DLS, offering additional advantages, such as estimation of particle concentration. NTA was performed using ZetaView^®^ (Particle Metrix, Ammersee, Germany). Just prior to the measurement, samples were diluted 5 000 000 times in MilliQ water or PBS, and 1 ml of the sample dilution was introduced into the instrument chamber using 1 ml syringes. To visualize the particles, a wavelength of 660 nm was used. Reported values of particle size and concentration represent averages of 3 separated recordings of 1 cycle at 11 positions.

#### Transmission Electron Microscopy (TEM)

To visualize the inner structure of the samples, while serving as an orthogonal sizing technique, TEM was performed, using a TFS Morgagni™ 268 microscope (FEI Company, Hillsboro, USA). Prior to the measurement, samples were diluted at 1:500 *v/v* ratio, in MilliQ water, and stained with uranyl acetate as contrasting agent. Carefully, 1 droplet (4 µl) of the sample dilution was deposited on Formvar support film hexagonal 200 mesh Cu grid (Electron Microscopy Sciences, Hatfield, UK) - previously treated with glow discharge. Grids were handled with special care, held by tweezers, so that the support film on the grids remained intact. Sample was left to adsorb for 1 min. In the meantime, 2 droplets of water (2 × 40 µl) and 2 droplets of uranyl acetate 1% solution (2 × 40 µl) were placed on a piece of the Parafilm^®^. The grid with the sample was afterwards immersed in a water droplet, blotted with filter paper, immersed in the second water droplet, and blotted again with filter paper. Finally, the grid was immersed in the first droplet of uranyl acetate for 15 s, blotted with filter paper, and then immersed in the second uranyl acetate droplet for 45 s and blotted in order to dry. The staining procedure was performed for each sample dilution. All grids were placed on a piece of filter paper in a Petri dish and left overnight. Samples were analyzed the following day.

#### pH measurements

The pH of the samples was measured at 25 °C, using the HI 9321 pH meter (Hanna Instruments Inc., Ann Arbor, MI, USA).

#### Zeta potential (ZP) measurements

In order to estimate the surface charge of the samples, measurements of the ZP were performed, assessing the electrophoretic mobility of the dispersed nanodroplets (Zetasizer NanoZS; Malvern Instruments, UK). Prior to the measurement, samples were diluted in 0.1 phosphate buffered saline (PBS) at a ratio of 1:500 (v/v), in order to provide adequate conductivity.

#### Estimation of nanoparticle density and size distribution by analytical ultracentrifugation (AUC)

The AUC analysis was conducted using a Beckman Coulter ProteomeLab™ XL-I (Brea, CA, USA) analytical ultracentrifuge. Prior to the measurement, samples were diluted in MilliQ water or heavy water - D_2_O, respectively, at the ratio of 1:1000 (v/v). The loading volume of the test sample cell was 390 µl, while the reference cell contained 400 µL of the diluent (water or heavy water). The sample holders were placed in an 8-hole rotor. Measurements were run at a constant rotation speed of 10,000 rpm, at room temperature. Interference and absorbance (290 nm) signals were collected in parallel (200 scans). Migration velocity of the droplets dispersed in two continuous phases of different densities was determined following the strategy described in the ISO standard ISO 18747-2 [39], which enabled determination of droplet density. To fit the experimental data, the *Sedfit* software was used (the ls-g*(s) model) [40]. Sedimentation coefficient distributions were calculated and successively transformed to mass-based size distributions using the “transform s distribution to r distribution” option in Sedfit.

#### Asymmetric flow field flow fractionation (AF4)

The Asymmetric Flow Field-Flow Fractionation (AF4) method was employed to assess the interactions of the nanoemulsions with the cell culture media. Samples were diluted in PBS and in cell culture media (500 times). After incubation at 37 °C during 6 h, separation was performed using a Postnova AF2000 AF4 system (Postnova Analytics, Germany) equipped with UV and multi-angle light scattering (MALS) detectors. The analysis was conducted over a total duration of 30 min. Injected sample volume was 100 µl. An amphiphilic membrane made of modified regenerated cellulose with a 10 kDa molecular weight cut-off was utilized. MilliQ water was used as the mobile phase, ensuring optimal separation. The specific flow rates and times for each step of the analysis are presented in Table 2. To study potential interactions, elution times of each sample were monitored and compared, and the radius of gyration (Rg) was determined.

**Table 2:** Separation steps and flow rates of the AF4 method

Focus step	Injection Flow	0.50 ml/min	2 min
	Cross flow - constant	3 ml/min	2 min
	Focus flow	3 ml/min	0.5 min
Elution step	Constant cross flow	3 ml/min	15 min
	Linear cross flow	3 − 0 ml/min	5 min
	Constant cross flow	0 ml/min	5 min
Rinse step	Tip pump	0.1 ml/min	0.5 min
	Focus pump	0.1 ml/min	
Detector flow during the analysis	0.5 ml/min		

#### Tonicity assessment

Tonicity of the formulations, as one of the important characteristics of parenteral dosage forms, was determined using semi micro osmometer Knauer K-7400 (Knauer, Germany), through the determination of the freezing point of the sample.

### Cell assays

#### Cells

The cell line selected for the experiments was RAW 264.7 (murine macrophages; American Type Culture Collection, Manassas, VA, USA). Cells were cultivated in the medium containing 10% of foetal bovine serum, 4.5 g/l glucose, supplemented with sodium-pyruvate, and 1% of penicillin and streptomycin. For all experiments, initial seeding density was 10^6^ cells/ml, so that the cells were confluent in the well plate after 24 h.

#### Estimation of the nanoparticle fraction reaching the cellular monolayer (“cellular dose”)

**Fig. 1:**
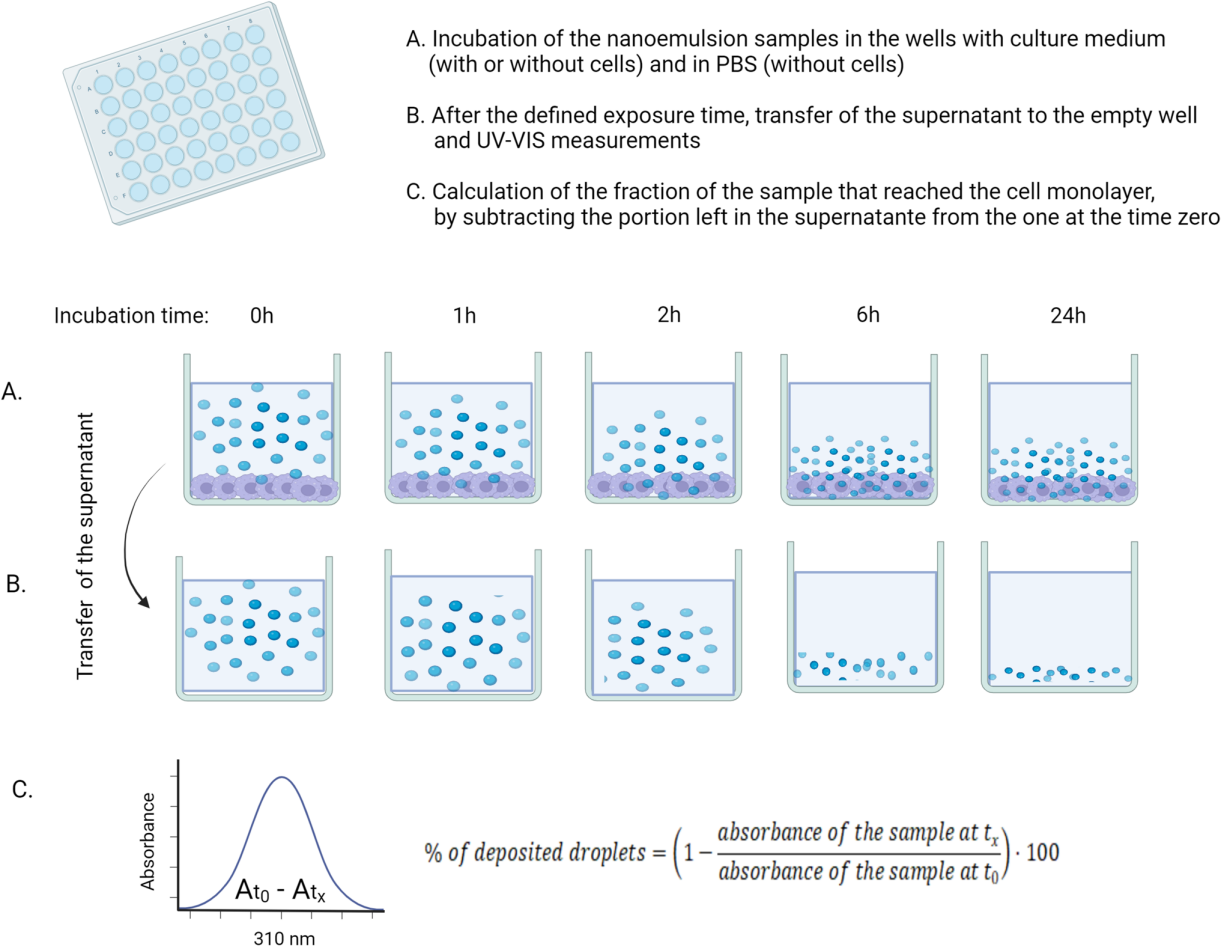
Graphical presentation of the protocol for assessing the fraction of the nanodroplets reaching the bottom of the well/cell monolayer (Generated by BioRender)

These measurements were performed at several time points (t_x_) for all the tested formulations that were incubated in the presence of cells or in their absence (in the cell culture media and in PBS), applying the procedure depicted in the Fig. 1. Based on the spectral analysis, 310 nm wavelength was selected as the detection wavelength. First, selected dilutions of the test samples were incubated under 3 different conditions: (1) with cells; (2) without cells, but in the cell culture media; (3) without cells in phosphate buffered saline (PBS). After the incubation time (1 h, 2 h, 4 h, 6 h and 24 h, at 37 °C), the absorbance of the supernatant was measured at 310 nm using the BioTek Synergy Mx microplate reader (BioTek instruments, VT, USA), and the portion of particles/droplets that reached the bottom/cell monolayer was calculated as follows (Eq. 1):

1 $$\%\:of\:deposited\:droplets=\left(1-\frac{absorbance\:of\:the\:sample\:at\:{t}_{x}}{absorbance\:of\:the\:sample\:at\:{t}_{0}}\right) \times 100$$

Cells (RAW 264.7) were seeded 24 h before the experiment, in order to attach to the bottom and reach confluency (initial seeding density of 10^6^ cells/ml).

#### WST-1

This assay is based on the evaluation of the mitochondrial activity, which is afterwards correlated with cell proliferation and viability. Cells were seeded in a 96-well plate (100 µl of the cell suspension, 10^5^ cells/100 µl) 24 h before the experiment. The day after, dilutions of the test samples were prepared and in the test wells medium was replaced by the appropriate sample solution. As positive control Triton X-100 0.1% solution was used, while culture medium served as a negative control. Cells were afterwards incubated for 6 h and 24 h at 37 °C. After incubation, the supernatant was moved to another cell plate (further used for the LDH assay), replaced by WST-1 reagent ((4-[3-(4-Iodophenyl)−2-(4-nitro-phenyl)−2 H-5-tetrazolio]−1,3-benzene sulfonate), Roche, Germany), followed by the incubation period of 2 h at 37 °C. WST-1 is metabolized by mitochondrial succinate-tetrazolium-reductase system to formazan, which is visible as a dark red colour in the well. The amount of formazan dye produced was calculated by measuring the absorbance at 440 nm, subtracting the background absorbance at 690 nm, using the BioTek Synergy Mx microplate reader (BioTek instruments, VT, USA). Cell-free culture medium with the corresponding concentrations of the nanoemulsions was used as the blank for the test wells, while for the positive and negative controls culture medium containing Triton X-100 (0.1%) or plain culture medium were used, respectively. Mitochondrial activity was calculated as follows (Eq. 2):

2 $$Mitochondrial\:activity\:\left(\%\right)=\:\frac{(sample\:absorbance-corresponding\:cell\:free\:blank\:absorbance)}{mean\:media\:control\:absorbance} \times100$$

#### Lactate dehydrogenase (LDH) assay

LDH assay represents a biologically relevant method for detecting cellular damage since it is based on the detection of lactate dehydrogenase – a cytoplasmatic enzyme that is released from the cells after loss of membrane integrity. Therefore, it is a direct measure of cellular integrity. Since LDH is detected in the cell supernatant, the same “batch“ could be used for both WST-1 and LDH, which was done in this case. After the cells had been incubated with the different nanoemulsion dilutions, the supernatant was transferred to another well and used for the analysis. For this purpose, LDH assay kit (ab65391, Abcam, The Netherlands) was used. The reagents were reconstituted and mixed following the instructions in the user manual, and 100 µl of the reaction mixture was added to each well (already containing 100 µl of the cell supernatant). The well plate was protected from light, shaken for 1 minute on an orbital shaker, and left for 10 minutes at room temperature. The absorbance was read at 490 nm, subtracting the background absorbance at 680 nm, using the BioTek Synergy Mx microplate reader (BioTek instruments, Vermont, USA). The percentage of the LDH leakage was then calculated applying the following equation (Eq. 3):

3 $$\varvec{\%}\:of\:the\:total\:LDH\:leakage=\:\frac{\left(sample\:absorbance-cell\:free\:sample\:blank\:absorbance\right)-mean\:media\:control\:absorbance}{(mean\:\:positive\:control\:absorbance-mean\:media\:control\:absorbance)} \times 100$$

In brief, the higher the percentage of the LDH leakage, the higher the toxicity.

#### Propidium-iodide (PI)-based assay

PI assay was applied as the fluorescence-based counterpart for toxicity testing. This compound can enter directly only into dead cells and attach to genetic material. Therefore, it can be used to distinguish dead from live cells. The assay was performed in 96-well plates, applying the same positive and negative controls as for the previously described in vitro assays. After the incubation, cell supernatant was removed and replaced with fresh cell media. Afterwards, to each well 8.2 µl of the propidium-iodide solution (Propidium Iodide Ready Flow™ Reagent, Thermo Fisher Scientific, MA, USA) was added. After 15 min of incubation (protected from light), fluorescence was measured applying the BioTek Synergy Mx microplate reader (BioTek instruments, VT, USA), at the excitation/emission wavelengths of 535/617 nm. The percentage of dead cells was calculated as follows (Eq. 4):

4 $$\%\:of\:dead\:cells=\:\frac{test\:sample\:fluorescence-negative\:control\:fluorescence}{positive\:control\:fluorescence-negative\:control\:fluorescence} \times 100$$

Finally, the number of live cells was estimated as the difference between 100% and percentage of dead cells.

### Statistical analysis

Whenever applicable, results were presented as the mean value of at least 3 repetitions ± standard deviation. Data treatment and statistical analysis were performed using the GraphPad Prism 9.5.1 software. After assessing the normal distribution of the data, comparison among the groups was assessed applying one-way ANOVA, followed by Tuckey post-hoc test. Comparison of 2 groups of data was done applying Student t-test. Statistical significance was defined as a p-value of less than 0.05.

## Results and discussion

### Physicochemical characterization: size and morphology

Prior to starting any in vitro experiment, all nanoemulsions were first characterized by applying several physicochemical techniques that would give an insight into their structure. This step is of high importance as biological properties of a nanostructured sample cannot be separately described from their physicochemical properties, since these two branches of characterization need to meet in order to correctly interpret the in vitro data [36]. This necessity has also been highlighted by Keck and Müller [41], who proposed a nanotoxicological classification system based on the size, biodegradability, and biocompatibility of a nanostructured sample. However, the comprehensive list of required physicochemical parameters for proper analysis is much longer and depends on the administration route, as well as the type of particles involved and their composition (simple drug delivery systems or highly complex, multicomponent, multifunctional structures) [42].

**Fig. 2:**
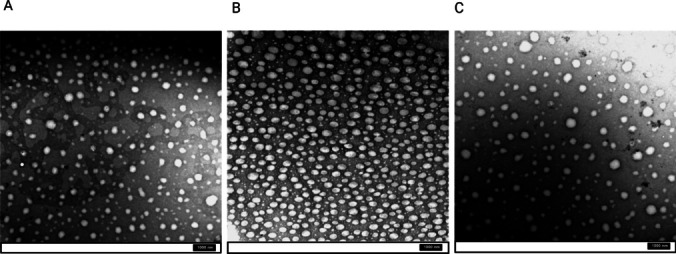
TEM micrographs of the samples at 14,000X magnification: NonPEG (**A**), P21 (**B**) and P51 (**C**)

As presented in the Table 3, particle size expressed as Z-average was around 100 nm across all samples, with significant differences depending on the diluent used. Specifically, samples exhibited larger Z-average values in PBS, which was particularly pronounced in the absence of PEG coating. The similar trend was observed with the PDI values. However, it can be concluded that all the three samples exhibit narrow size distributions. Taking a closer insight into the particle shape and size, TEM micrographs revealed the existence of at least 2 populations of spherical droplets (Fig. 2): the dominant one (minuscule droplets, below 100 nm), and a population of bigger droplets, still below 500 nm. Finally, NTA corroborated these findings, affirming a size spectrum ranging from 50 to 250 nm based on volume-based distribution, consistent with a median slightly surpassing the 100 nm threshold (Table 4). What is evident is that the particle concentration, estimated based on NTA results was higher for PEGylated samples, compared to the nonPEGylated ones. This may be due to the already known inherent variability of the NTA for this specific type of analysis [36]. Alternatively, it might be due to the presence of additional structures formed of PEG chains that were not attached to the particle surface.

**Table 3:** DLS sizing results of the test samples

Sample	Diluent	Dilution	Z-average (nm)	PDI	Intercept of the correlation function	Attenuator
NonPEG	Water	1:500 (*v/v*)	91.70 ± 0.40	0.060 ± 0.009	0.937	6
	PBS		101.70 ± 1.19	0.153 ± 0.017	0.920	6
P21	Water		89.70 ± 0.59	0.062 ± 0.014	0.939	5
	PBS		92.71 ± 0.41	0.072 ± 0.019	0.967	4
P51	Water		96.00 ± 0.52	0.079 ± 0.011	0.964	5
	PBS		102.60 ± 0.77	0.103 ± 0.017	0.964	5

*Z-ave *intensity-based average hydrodynamic diameter, *PDI *polydispersity index, *N* = 3, *n* = 10

**Table 4:** Results of the sizing experiment performed by NTA 500·10^6^/*N* = 3

Sample	Diluent	Dilution	Median (nm)	D10	D50	D90	Particle concentration (particles/ml)
NonPEG	Water	1:5·10^6^(*v/v*)	109.20 ± 0.81	58.37 ± 1.34	96.23 ± 0.47	156.77 ± 2.36	4.3 ± 0.3·10^14^
	PBS		112.70 ± 2.97	54.45 ± 3.18	107.95 ± 6.29	225.15 ± 11.53	
P21	Water		104.23 ± 0.49	50.23 ± 0.67	92.87 ± 0.81	182.53 ± 2.30	5.4 ± 1.2·10^14^
	PBS		111.9 ± 3.82	51.95 ± 2.05	112.10 ± 6.22	252.55 ± 6.58	
P51	Water		106.23 ± 1.00	46.43 ± 1.29	95.67 ± 0.87	186.87 ± 4.98	6.13 ± 0.47·10^14^
	PBS		110.8 ± 1.6	53.4 ± 2.17	103.5 ± 5.97	218.9 ± 5.07	

*D10 *mean hydrodynamic diameter at 10% of the particle volume, *D50 *mean hydrodynamic diameter at 50% of the particle volume, *D90 *mean hydrodynamic diameter at 10% of the particle volume

Apart from similar size distribution, all samples were characterized by a negative zeta potential (Table 4). In general, higher absolute values of zeta potential are coupled with better long-term stability. In particular, if the dominant stabilisation mechanism is electrostatic, absolute values of zeta potentials above 30 mV are considered an indicator of good long-term stability [43, 44]. However, these values cannot be taken strictly, especially when stabilisation is provided by the use of steric stabilizers or a combination of electrical and steric effects. Due to the presence of steric stabilisers and PEG coating during the measurement of the zeta potential, the diffuse layer does not move along with the particle in the electric field, so the shear plane is shifted, and consequently, zeta potential is measured at a greater distance from the Stern layer. Due to the exponential decay with increase in the distance from the Stern layer, measured values are significantly lower compared to a system that does not contain steric stabilizers [45]. In addition, positively charged nanoparticles tend to interact more with the cells (due to the opposite charge of the cell membrane), but consequently negatively charged systems are expected to be less toxic. Recent studies indicate that surface charge density is a more effective predictor of toxicity than net charge (zeta potential) [46].

**Table 5:** pH, zeta potential and osmolarity of the nanoemulsion samples (*N* = 3, *n* = 3)

Sample	pH	Zeta potential (mV)	Osmolarity (mOsm/l)
NonPEG	6.88 ± 0.02	−14.1 ± 1.7	305 ± 2
P21	6.89 ± 0.01	−16.1 ± 0.6	306 ± 1
P51	6.77 ± 002	−13.2 ± 0.7	298 ± 3

Taken all together, all three samples exhibit similar physicochemical properties, they were isotonic and with the pH value slightly below 7 (Table 5). What is interesting is that the PEG coating did not affect the size, even though such an effect could have been expected.

However, the most important property that represents a milestone for further investigation is depicted in the safety profile. In vitro safety assessment precedes any other biological investigation. Therefore, these aspects have also been investigated with attention, aiming to establish a link between physicochemical properties of nanosystems with their in vitro performance.

### In vitro biological assessment

#### Assessing the fraction of the samples reaching the cell monolayer

In in vitro assays, nanosystems exhibit unique behaviour compared to the simple molecules. This is due to their shape, density, surface charge and other properties influencing their interaction with their surroundings. Therefore, usual in vitro toxicity assays ought to be adapted to these more complex systems. In order to establish the optimal conditions for this type of assays, an experiment was designed with the idea to estimate the fraction of the nanodroplets reaching the cell monolayer. Such an approach would facilitate the selection of a proper incubation time. In addition, it was interesting to see if there is any difference between the PEGylated and nonPEGylated formulations.

**Fig. 3:**
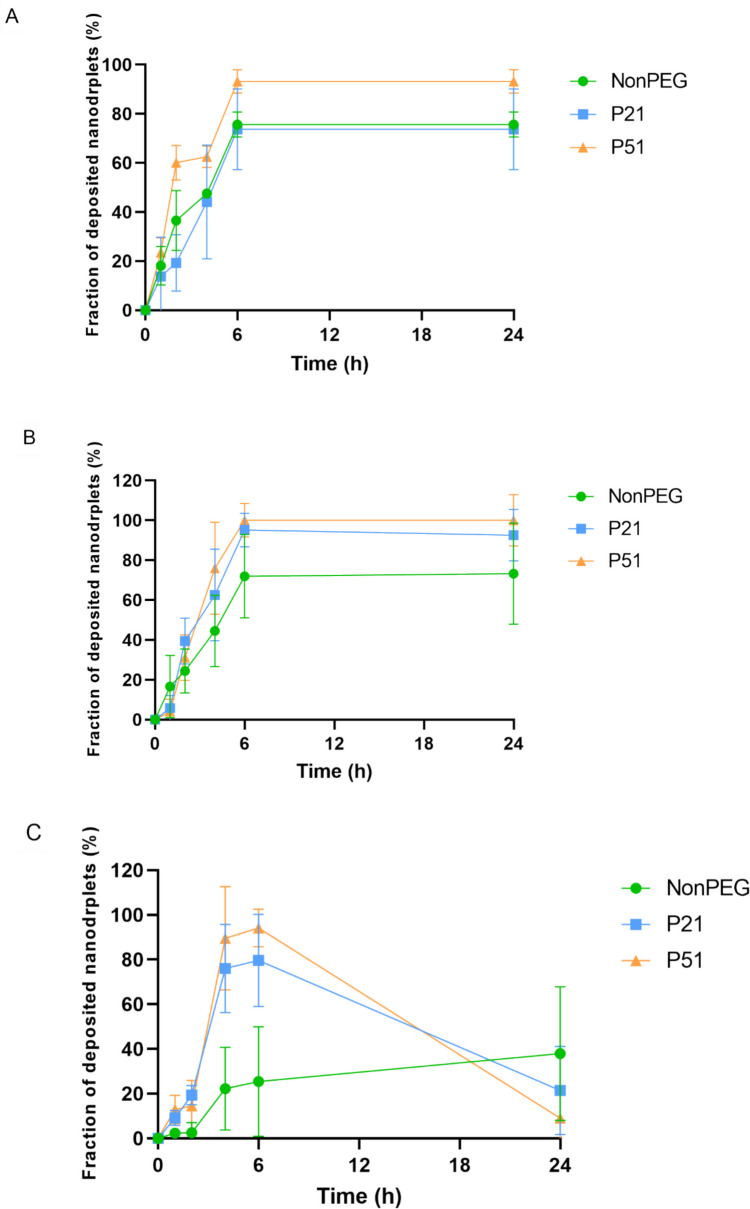
Fraction of the nanoformulation reaching the cell monolayer/bottom of the well: wells containing cells in the culture medium (**A**); wells without cells containing culture medium (**B**); wells without cells containing PBS (**C**) (*N* = 3, *n* = 3)

As depicted in Fig. 3, there is a time-dependent adsorption of the nanodroplets regardless of the experimental conditions (with or without cells). When samples are incubated in the presence of cells and in the culture medium, corresponding to the experimental setup of the in vitro experiments, samples were able to reach the cell monolayer to the highest extent after 6 h of incubation. However, the maximal extent was different: around 90% of sample P51 came in touch with the cell monolayer, while for the other 2 samples, it was around 75%. The portion of the droplets that were reaching the cells did not change with time (after 24 h). Similarly, when samples were incubated in the cell culture media only (absence of cells Fig. 3B), the trend was the same, but P21 and P51 were now both far more efficient with respect to the nonPEGylated nanoemulsion. Interestingly, when the incubation was done in PBS only (Fig. 3C), the difference was even more pronounced. However, this was not the only dissimilarity. It is noteworthy that after longer incubation times, the portion of the droplets reaching the cell monolayer was low. We may hypothesize that droplets migrated away after having reached the bottom, driven by a density slightly lower than that of the medium. It may be supposed that in the cell culture medium proteins and other biomolecules attach to the surface of the droplet, directing them towards the cells. This may be the reason for better performance of every sample in the culture medium (especially in the presence of the cells) compared to the simplified surrounding, represented by PBS. Similar findings were reported by Richtior and coworkers [1]. However, in the literature there are only scarce examples of this kind of experimental investigations. This should be particularly important in the in vitro testing of lipid-based nanosystems (e.g., in dose calculation), due to their possible lower density compared to the inorganic or polymeric nanoparticles. Still, there are reported findings related to iron-based nanoparticles, where similar problematics and strategies to overcome them have been discussed [47].

Having in mind the different behaviour of the investigated samples in terms of their ability to reach the bottom of the well, and with the idea to be more certain about the conclusions, additional experiments were performed applying the AUC. This segment enabled supplementary sizing estimations, with calculation of the density of the droplets [48]. While the AUC method is commonly used for protein size analysis and kinetic investigations, its utility extends to measuring particle size, reaching the micrometre scale, contingent upon the nanoparticles’ density. This versatility has been acknowledged by researchers in both protein and particle size analysis studies [36, 48–53].

Since there was no significant difference in terms of size, it could be reasonable to conclude that density difference combined with surface characteristics represent the key driving forces for contrasting behaviour among the samples, especially in the context of the surrounding liquid composition (culture medium vs. PBS).

**Fig. 4:**
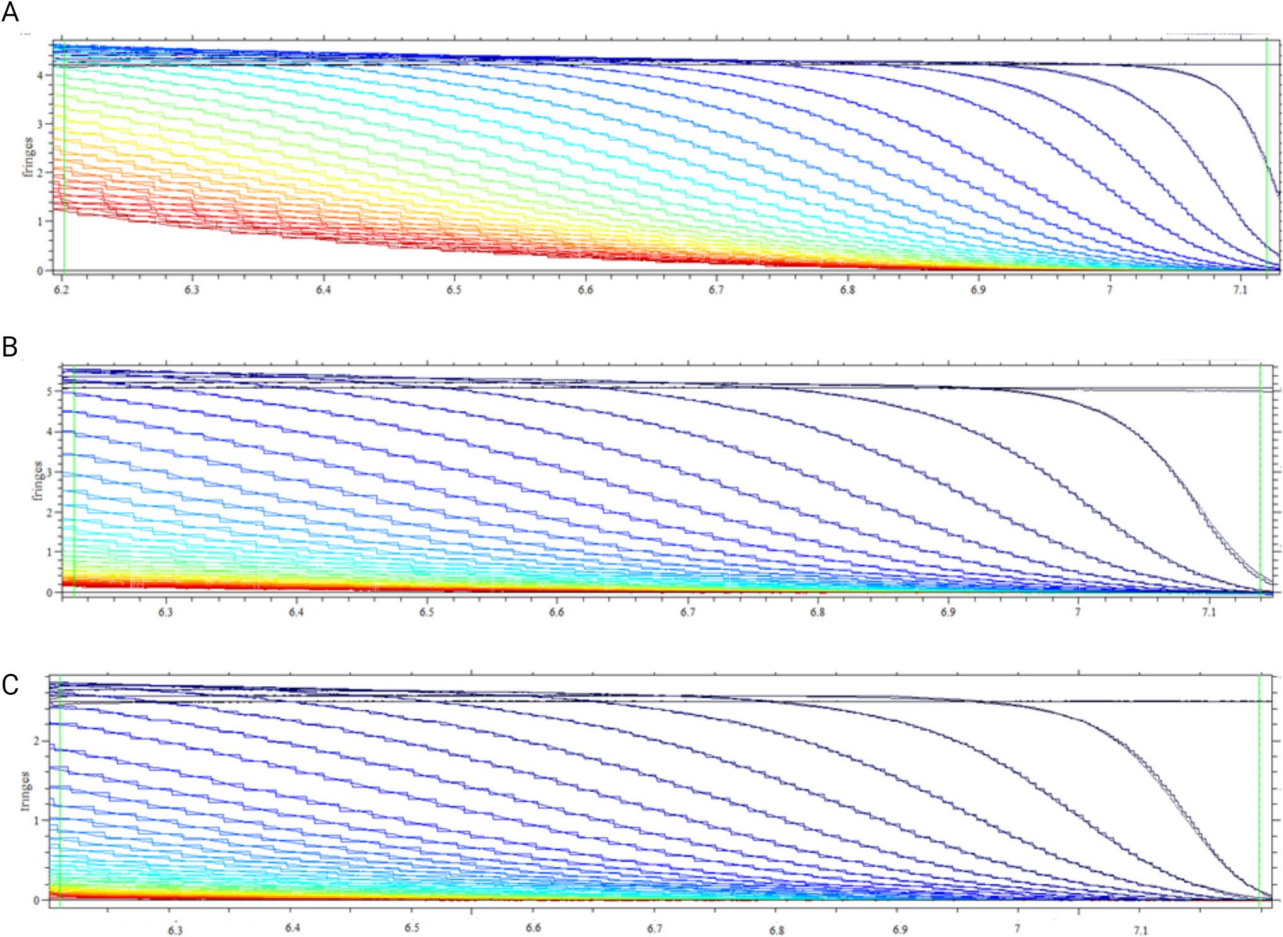
AUC sedimentation profiles of NonPEG (**A**), P21 (**B**) and P51(**C**) diluted 1000 times in D_2_O. Y-axis represents interference data, while the X-axis represents radial positions (distance in cm from center of rotation) in the sample cell. The changing colour of the curves from dark blue to red corresponds to increasing centrifugation time (*N* = 3. *n* = 3)

**Fig. 5:**
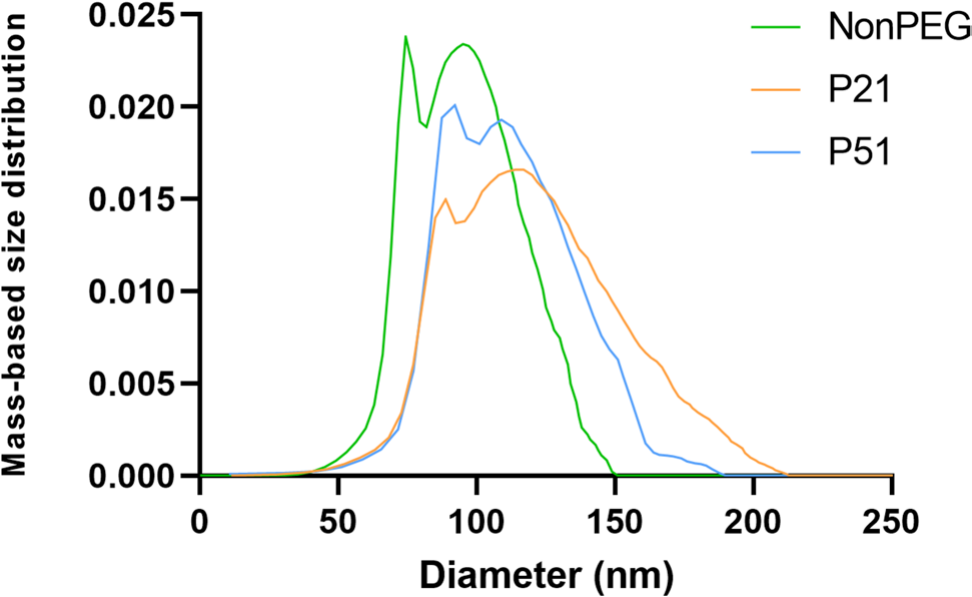
Size distribution of the nanodroplets of the samples NonPEG, P21 and P51 determined by AUC (*N* = 3, *n* = 3)

The “inverted“ sedimentation was visible at the first glance after the AUC (Fig. 4). Namely, during the centrifugation, the droplets were not undergoing sedimentation, but were floating, due to their density being lower than water. Data analysis resulted in the following density values: 0.962 g/ml (NonPEG), 0.970 g/ml (P21), 0.972 g/ml (P51). Particles without PEGylation (NonPEG) showed the lowest density value, while particles with thicker PEG coating (P51) the highest. Concerning size distributions, they were in good accordance with the data obtained by TEM, since 2 peaks were detected (Fig. 5).

**Fig. 6:**
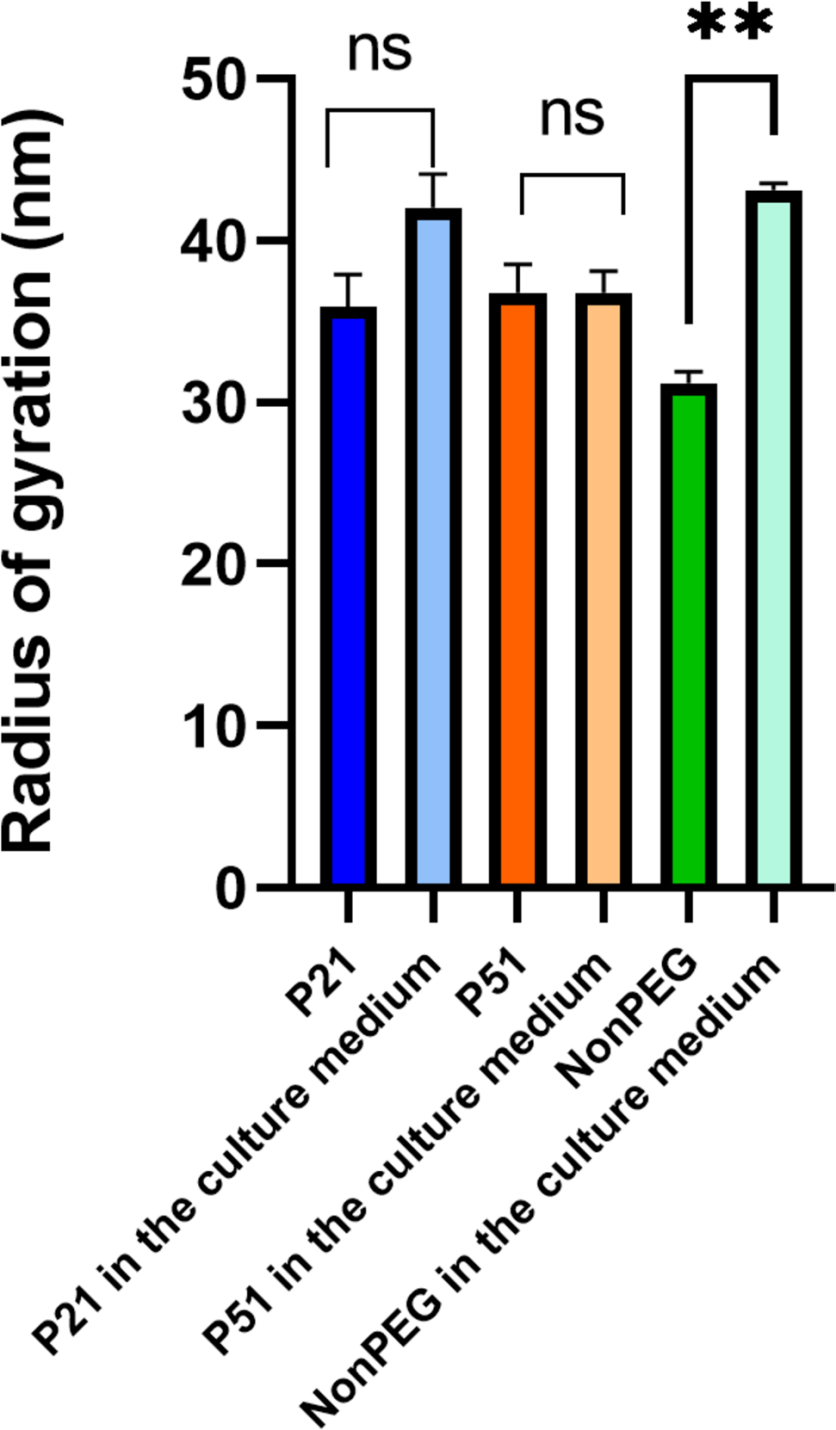
Rg values for the three tested nanoemulsions after dilution in PBS and in cell culture medium (*N* = 3, *n* = 3; **p < 0.01)

**Fig. 7:**
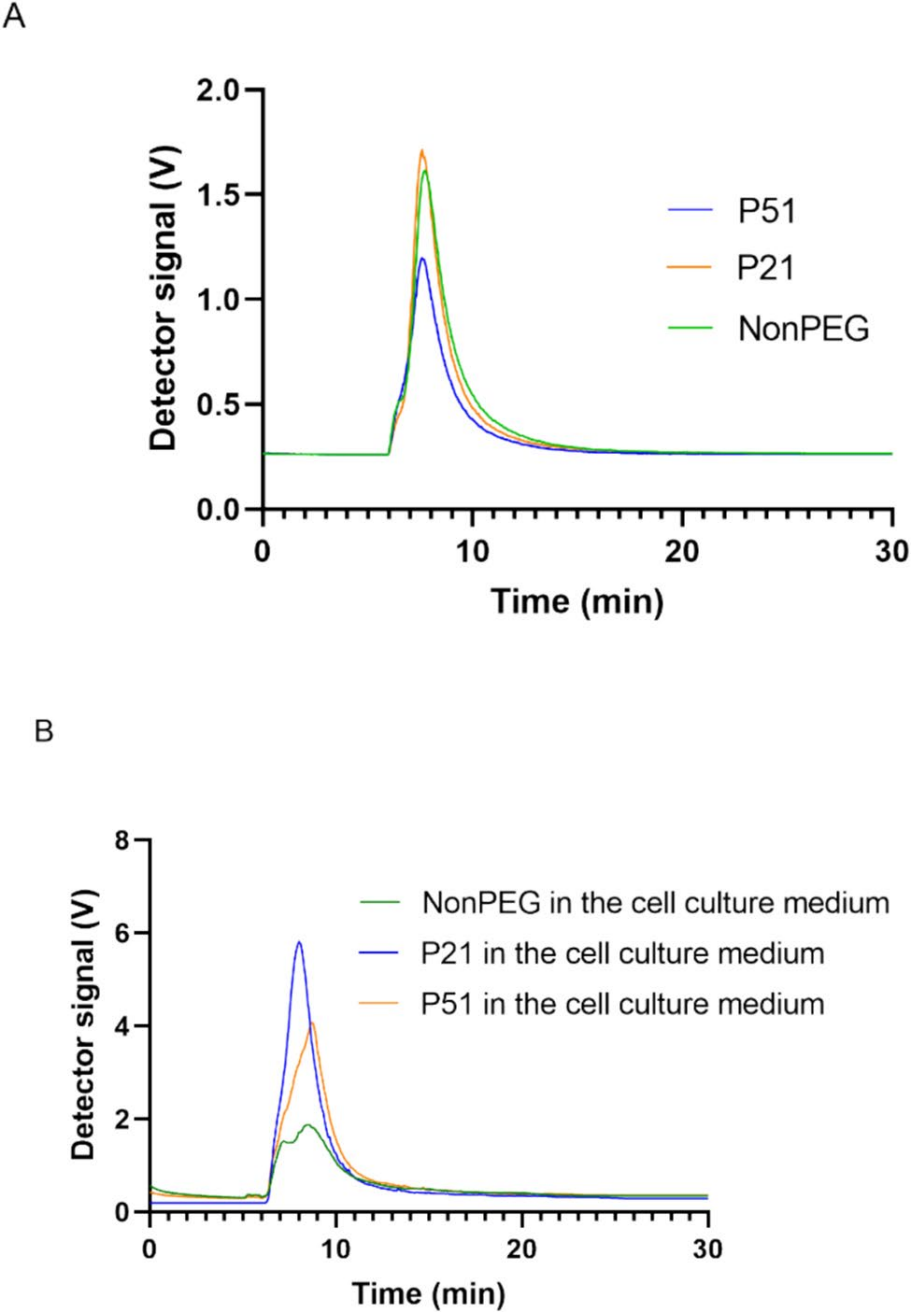
AF4 analysis of the NonPEG, P21 and P51 in PBS (**A**) and after 6 h incubation in the cell culture medium at 37 °C (**B**) (*N* = 3, *n* = 3)

Based on these findings, it was confirmed that nanoemulsion droplets might need longer time to reach the cell monolayer than liposomes, polymeric or inorganic nanoparticles due to their low density and their tendency to float. Most probably, in the culture medium, proteins from the surrounding medium attach to the droplets, they dynamically interact with the droplets, modifying their effective density, facilitating the transport toward the cells. Even more, cells secrete various molecules that may also contribute to the overall affinity of the nanodroplets to the cell monolayer. In contrast, when samples were incubated in PBS, it was obvious that they did not have the tendency to reach the bottom of the well, most probably because there were no biomolecules present. To support this hypothesis, AF4 analysis was performed on the samples that were diluted in the same matrices (PBS and cell culture media), they were incubated at 37 °C, for 6 h, followed by the determination of the Rg. It is known that Rg is related to the mass density of particles and changes when additional biomolecules, such as proteins, adsorb onto the particles. This technique is considered the method of choice for particle sizing and interaction studies in complex environments, especially when combined with detectors like dynamic light scattering (DLS) or multi-angle static light scattering (MALS) [54]. Obtained Rg values (Fig. 6) correspond to the mass density of the nanoemulsion droplets estimated by AUC (NonPEG < P21 < P51). After incubation with the cell culture medium, the Rg increased significantly for the NonPEG sample, while P21 and P51 did not show significant differences, indicating interactions between biomolecules in the culture medium and the non-PEGylated formulation. Furthermore, the AF4 signal analysis revealed that only the eluogram shape of the non-PEGylated sample changed significantly (Fig. 7). This indicates that, for this sample, the presence of the cell culture medium was not additive (the detector response did not increase quantitatively, but the shape of the curve changed) unlike with PEGylated formulations, proving that proteins and other biomolecules from the medium interacted with the NonPEG. Some interactions were observed with the other two samples, but they were less prominent.

The findings suggest that the adsorption of biomolecules from the culture medium enhances the likelihood of NonPEG nanoemulsion droplets reaching the cell monolayer, attributed to the increase in mass density. This conclusion aligns with the AUC density values, which explains the observed differences in behavior between complex media (cell culture medium) and simpler solutions like PBS. Although NonPEG formulation exhibits the lowest intrinsic density, its exposure to a complex matrix, such as one containing proteins, promotes significant biomolecule attachment to the droplet surfaces. This phenomenon occurs to a greater extent compared to PEGylated formulations, where the presence of a steric PEG layer likely reduces biomolecule adsorption. Consequently, this increased biomolecule attachment explains the differences in the droplets’ ability to interact with the cell monolayer, highlighting the crucial role of the surrounding medium’s complexity in influencing nanoparticle behavior. Moreover, it has already been discussed that the microenvironment, such as the extracellular matrix, various microenvironmental factors, and pH, can also change sample properties and affect their interactions with the cells, and potentially, cellular internalization [55].

Finally, these observations can be additionally supported by the obtained density values. Since the samples exhibited densities lower than the surrounding, present proteins and other biomolecules that could attach to the surface are able to increase effective particle density and direct them towards the bottom of the wells. In addition, NonPEG sample is supposed to have a higher affinity to biomolecules than PEGylated ones (which was proved through the AF4 analysis). In the presence of cells, this leads to a behaviour very similar to P21. In spite of their lower density, NonPEG nanodroplets were leaving the supernatant with similar speed to the theoretically higher density P21 nanodroplets. Without cells, their behaviour is more distant and follows more the pattern of the density difference.

Interestingly, when cell culture medium was used, no difference was observed between 6 h and 24 h of incubation.

Taken all together, it could be suggested that, in this case, the minimal incubation time needed for a reliable in vitro toxicity assay should not be less than 6 h. What should be also considered is the fact that even after 24 h, the fraction of the sample that reached the cells did not increase, while only the sample with the highest density (P51) reached the cell monolayer completely. Such an observation clearly demonstrates that we cannot consider that nanoparticulate dispersions will behave in the same manner as solution of small molecules in the in vitro biological assays. What is more, nanoparticulate samples of the same type (shape, composition) differ with respect to size, density, and surface properties, which can also affect the adsorption of surrounding molecules, leading to different in vitro behaviour.

#### Toxicity assays: WST-1, LDH and PI-based assay

Considering the results of the previous experimental segments, cell toxicity assays were performed ta 2 incubation times of 6 h and 24 h, and results were compared.

Since nanoparticles scatter light or even interact with the reagents, consequently compromising the reliability of the results, experiments were designed in a way that this issue was fully eliminated. This considers that for each test sample concentration, an appropriate control was prepared that could subtract scattering or absorption of the sample (which is concentration dependent). Accordingly, the appropriate cell plate design was used (Supplementary material section). Applying such a design it is possible to test 2 samples at 8 dilutions, each in triplicate. The top and bottom edge of the plate served as blanks for each of the test concentrations (A2-A9), for negative (A10) and positive (A11) controls. During the data analysis, absorbance/fluorescence values from these wells were subtracted from the corresponding values obtained in the test wells. The first (1 A-H) and the last (12 A-H) column contained the cell culture medium only, and they were not used in the analysis, they mainly served to maintain the constant humidity around the cells that were seeded in the central part of the plate. Having the blank values for each test sample concentration provided the possibility to subtract any potential scattering effect of the nanodroplets, or potential interaction of the sample with the reagent. Since the test samples were “empty” nanoformulations, the test concentrations were presented as % of the sample that was used to treat the cells.

**Fig. 8:**
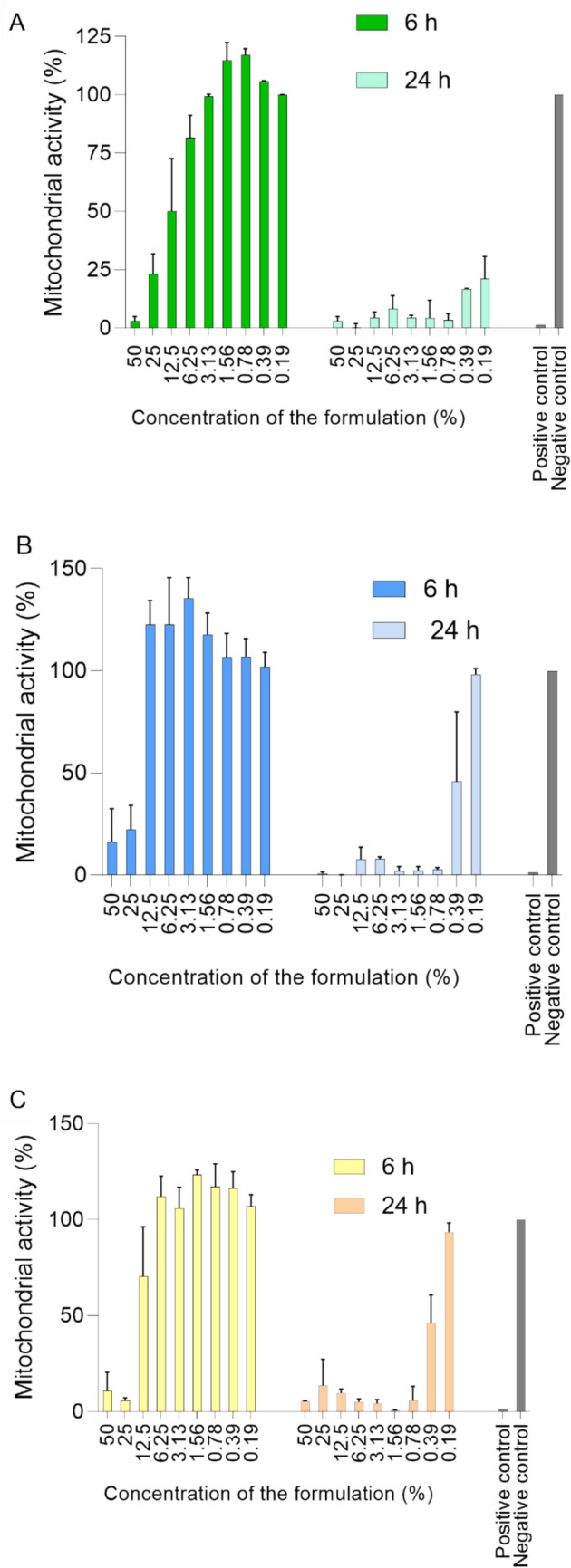
WST-1 toxicity assay results: mitochondrial activity (relative to the untreated cells) as a function of sample concentration after 6 h and 24 h of incubation (RAW 264.7): NonPEG (**A**), P21 (**B**) and P51 (**C**) (*N* = 3, *n* = 3)

First, WST-1 was performed, as one of the most commonly used assays of estimation of cell viability through the assessment of mitochondrial enzymatic activity. As depicted in the Fig. 8, after 6 h of the treatment, mitochondrial activity was increasing with decreasing sample concentration. Even though the first concentrations seemed to cause high cell damage, this should not be considered problematic since the range of concentrations started at a really high percentage of the sample at 50%. Surprisingly, after 6 h of the treatment, the nonPEGylated sample expressed higher toxicity compared to the P21 and P51, even though it was expected differently. However, after 24 h, mitochondrial activity was low for all test concentrations in the case of NonPEG, while P21 and P51 showed the expected trend in mitochondrial activity, where the two lowest concentrations tested may be considered as non-cytotoxic.

**Fig. 9:**
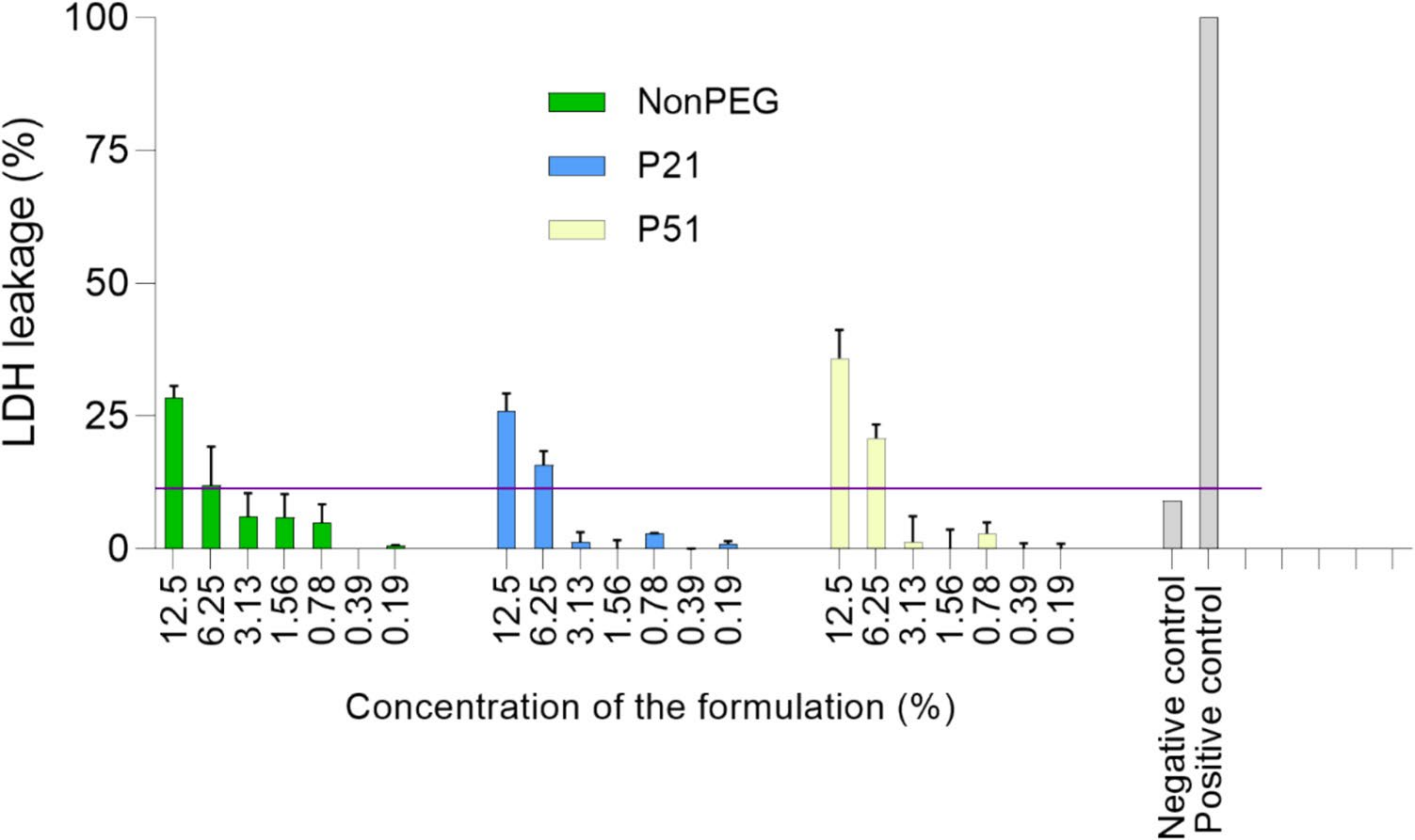
LDH assay results: enzyme leakage depending on the formulation concentration after 6 h of incubation (RAW 264.7; *N* = 3, *n* = 3); The horizontal line represents the 10% LDH leakage threshold

Even though this assay is commonly used, it is giving just a rough and indirect estimation of the cellular viability since it is not directly detecting cell damage. In order to address this problem, LDH test was performed. This was considered more appropriate since LDH is released when the cell membrane is damaged, representing cytotoxicity in a more realistic manner. Results confirmed the same trend – the lower the test concentration – the lower LDH leakage (Fig. 9). Even though there is no exact recommendation on the percentage of the LDH leakage considered as normal, it appears reasonable that results below 10% of the leakage could be considered as nontoxic. Moreover, nontreated control cells exhibited an LDH leakage baseline of around 9%. Comparing the results, it was difficult to capture the differences among the formulation, and after 24 h of the incubation, toxicity was very high for all the samples.

**Fig. 10:**
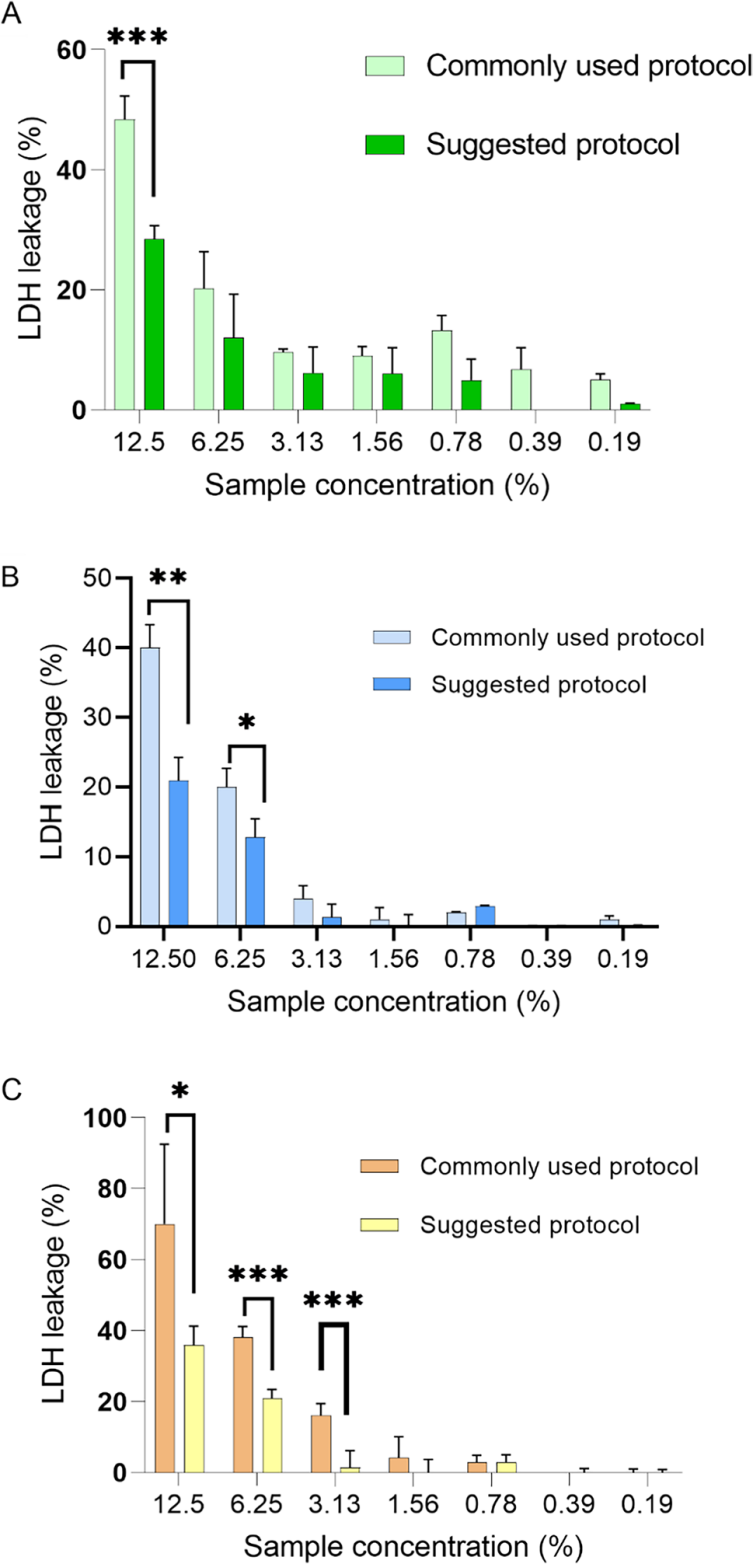
Comparison of the LDH assay results applying 2 ways of data analysis: commonly used one (with 1 blank for all the samples) and adapted one (with a corresponding blank for each sample concentration): NonPEG (**A**), P21 (**B**) and P51 (**C**); *N* = 3, *n* = 3. *** *p* < 0.001; ** *p* < 0.01; * *p* < 0.05

In this experimental segment the authors aim to highlight how nanoparticles can impact assay outcomes, particularly in an assay where the absorbance of cell supernatant is analyzed in the presence of the nanoparticles (unlike in the WST-1 assay where nanoparticles are removed). The presence of nanoparticles can lead to high absorbance values in the test wells, suggesting significant LDH leakage even at lower sample concentrations. Typically, ratio between the original and subtracted values was 2:1. However, by utilizing appropriate controls, it becomes clear that the actual results differ. By factoring out the scattering effect, the obtained values provide a more realistic assessment of toxicity. For instance, the calculation of LDH leakage using a standard toxicity protocol yielded values twice as high as those suggested in this study (Fig. 10). It is evident that a nuanced approach to characterizing nanomedicine must consider the specific characteristics associated with their particulate nature.

**Fig. 11:**
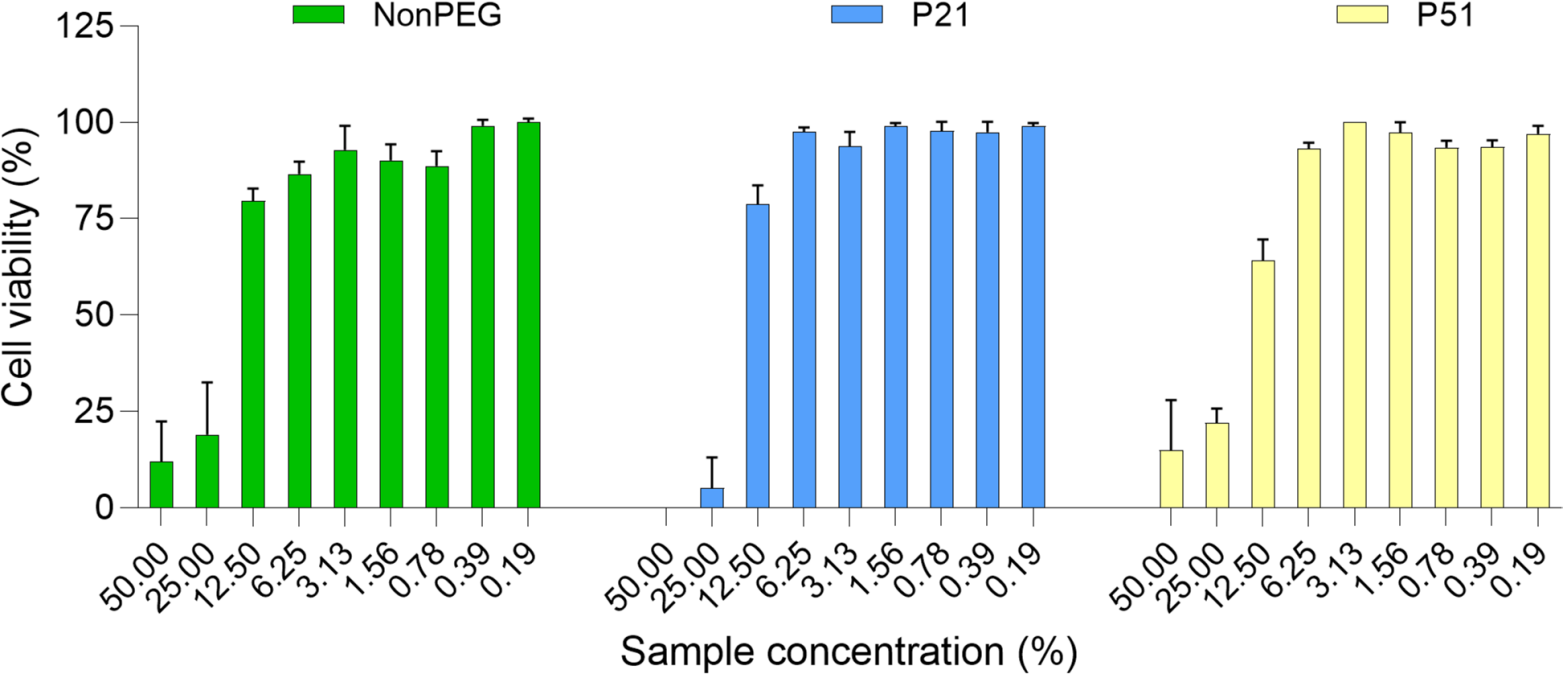
PI-based cell viability assessment; *N* = 3, *n* = 3

Finally, the last performed assay was the PI-based fluorescence assay, able to directly distinguish live from dead cells. This assay was performed only at the shorter incubation time (6 h), with the idea to better capture the differences between the formulations. However, results were similar to those obtained with the LDH assay – no specific difference was observed (Fig. 11). What is worth mentioning is the fact that PI can penetrate into the cells that have a damaged membrane, being suitable to detect necrotic cells in culture that lost membrane integrity [56]. Therefore, it could be nicely compared to the LDH assay in terms of the mechanism of the toxicity.

What is interesting is the fact that the WST-1 assay showed nonPEGylated sample to be more toxic compared to the PEGylated ones. If we refer to the sample adsorption assay (Fig. 3), this result is even more surprising. It could be hypothesized that, due to the smaller spatial volume of the nonPEGylated particles, it was easier for the nanodroplets to be internalized, disturbing the mitochondrial function, compared to the PEGylated ones. These results might also suggest that particle sedimentation/adsorption to the cell monolayer is not directly correlated to their uptake by the cells. Moreover, the PEG coating might affect the efficacy of nanoformulations to internalize into the cells by increasing the area per lipid, due to its interaction with the interfacial molecules and possible penetration in the lipid bilayers, changing the molecular dynamics, as described also by other authors [57]. In addition, the PEG coating (PEG “corona”) may decrease interaction with the cells compared to the nonPEGylated samples [58]. However, cell cytotoxicity is manifested in various types of cellular damage. Therefore, the proper assay method should be selected on the basis of the origin of the altered cellular condition (type of cellular damage). For example, samples causing cellular redox perturbation and mitochondrial dysfunction could easily be detected by WST-1, MTT or related assays. On the contrary, if the origin of the toxicity is different, this type of toxicity evaluation, even though easy and commonly applied, may not give adequate results. In addition, the LDH release assay is suitable for determining cytotoxicity that causes membrane damage but it is not suitable for determining the extent of toxicity with other underlying mechanisms [59]. This is especially important in detecting toxicity at early stages of shorter incubation times.

What could be suggested is that if the underlying mechanism for induced cellular disturbance is unclear, in vitro safety aspects should be determined using at least two experimental methods based on different principles. Additionally, proper blanks should always be taken into consideration due to the high probability of nanoparticle-reagent interactions or scattering effects of the sample, as is shown here.

Finally, in the presented case, it is clear that cell membrane damage was significant and similar in all cases, most probably due to the presence of sodium oleate. It has already been discussed that oleate is capable of inducing necrotic cell death in liver sinusoidal endothelial cells [60], and, among other factors, these effects could be caused by changes in membrane lipid composition and fluidity [61]. However, at appropriate concentrations, this effect may be considered as beneficial, due to the increased cell penetration and consequently more efficient delivery of the active molecules into the target cells.

LDH and PI-based assays were in good correlation, while WST-1 pointed out that nonPEGylated formulation altered the mitochondrial activity to a more pronounced extent compared to the PEGylated formulations. Further mechanistic studies should be performed to confirm and elucidate the origin of this effect.

## Conclusion

The aim of this study was to assess CQAs of parenteral nanoemulsion formulations by measuring several physicochemical parameters and linking them to their in vitro performance. The minimal, but most appropriate set of characterization should encompass size and size distribution through at least two orthogonal techniques, such as DLS and electron microscopy. Added value was obtained by additional analysis applying AUC, especially for lipid-based systems.

More importantly, prior to in vivo studies, it is crucial to perform an in vitro assessment of cytotoxicity. It is important to emphasize the need for well-designed control samples to account for nanoparticle scattering effects, as illustrated in this study through the LDH assays. To ensure an accurate assessment of cytotoxic effects, it is recommended to use at least two different assays, especially when the toxicity mechanism is unknown. Additionally, it is important to recognize that commonly used assays may not be all-encompassing, particularly when their outputs are not directly linked to cellular damage mechanisms. The authors also propose shorter incubation times for better analysis of the sample effects, although this should be cautiously considered, especially with lipid-based systems. Due to the assumed lower density of lipid-based samples compared to their environment, they require a certain amount of time to reach the cell monolayer and begin interacting with the cells, likely aided by the presence of proteins and other biomolecules.

To effectively translate preclinical in vitro data to in vivo applications, it is crucial to obtain reliable results. As mentioned, nanoformulations exhibit specific physicochemical characteristics that necessitate a careful approach and assessing bio-nano interactions. Even with robust in vitro data, nanoformulations often demonstrate distinct behaviour when transitioning from the controlled environment of cell cultures to the complex in vivo environment. This discrepancy can be attributed to factors such as protein corona formation, cellular uptake dynamics, biodistribution, and interactions with the immune system [62]. In the present study, critical quality attributes (CQAs) such as particle size, surface charge, and bio-nano interactions were examined and contextualized within the framework of in vitro toxicity using three distinct assays. It is important to interpret in vitro assay results in light of the physicochemical CQAs of the tested formulations. However, to better simulate real scenarios, these formulations should also be evaluated in more complex environments, such as human serum or plasma, with additional testing for immunogenicity and hemolysis.Basic size measurements using diverse techniques are essential, but more thorough understanding necessitates additional characterization, such as assessing particle-protein interactions, while standard static in vitro assays may be improved by utilizing a microfluidic setup. This comprehensive concept would offer further insights and help align conclusions more closely with real contexts, but, on the other side, may elevate analysis costs.

Given the complexity of the nanosystems, incorporating in chemico, in vitro, and ex vivo methodologies can provide valuable indications, although ultimate safety and efficacy determinations rely on in vivo models. Therefore, the authors advocate for a cost-effective strategy of combining well-designed physicochemical and biological assays in a standardized manner as an initial step in reliable preclinical characterization of nanomedicines, aiming to mitigate potential challenges. These efforts were undertaken with regulatory requirements in mind to ensure the quality, safety, and efficacy of nanomedicines.

## Supplementary information

Below is the link to the electronic supplementary material.

ESM 1 (DOCX 80.4 KB)

## Data Availability

The datasets generated and analysed during the current study are available from the corresponding author on reasonable request. They will also be available in the institutional repository.
