# Supplementary material for: When conventional approach in toxicity assays falls short for nanomedicines: a case study with nanoemulsions
Source: Drug Deliv Transl Res. 2025 Jan 8;15(8):2814–32. doi: 10.1007/s13346-024-01776-7 (PMC12213998; doi:10.1007/s13346-024-01776-7)
Supplement: Supplementary file 1 — (DOCX 80.4 KB) [file 13346_2024_1776_MOESM1_ESM.docx]

# Supplementary material

# When Conventional Approach in Toxicity Assays Falls Short for Nanomedicines: A Case Study with Nanoemulsions

Ines Nikolić^1,2^, Jelena Đoković^2^, Dora Mehn^3^, Giuditta Guerrini^3^, Snežana Savić^2^, Olivier Jordan^1^, Gerrit Borchard^1*^

^1^University of Geneva – Faculty of Science, Section of Pharmaceutical Sciences, Geneva, Switzerland

^2^University of Belgrade – Faculty of Pharmacy, Department of Pharmaceutical Technology and Cosmetology, Belgrade, Serbia

^3^EC Joint Research Center, Nanobiotechnology Laboratory, Ispra, Italy

*corresponding author

gerrit.borchard@unige.ch

### Estimation of the nanoparticle fraction reaching the cellular monolayer (“cellular dose“)

Table 5. Cell plate design used in the *in vitro* assays

|  | **1** | **2** | **3** | **4** | **5** | **6** | **7** | **8** | **9** | **10** | **11** | **12** |
| --- | --- | --- | --- | --- | --- | --- | --- | --- | --- | --- | --- | --- |
| **A** | Cell culture medium | **S1D1** | **S1D2** | **S1D3** | **S1D4** | **S1D5** | **S1D6** | **S1D7** | **S1D8** | **Negative  control** | **Positive control** | Cell culture medium |
| **B** | Cell culture medium | **Cells + S1D1** | **Cells + S1D2** | **Cells + S1D3** | **Cells + S1D4** | **Cells + S1D5** | **Cells + S1D6** | **Cells + S1D7** | **Cells + S1D8** | **Cells  + negative control** | **Cells  +  positive control** | Cell culture medium |
| **C** | Cell culture medium | **Cells + S1D1** | **Cells + S1D2** | **Cells + S1D3** | **Cells + S1D4** | **Cells + S1D5** | **Cells + S1D6** | **Cells + S1D7** | **Cells + S1D8** | **Cells  + negative control** | **Cells  +  positive control** | Cell culture medium |
| **D** | Cell culture medium | **Cells + S1D1** | **Cells + S1D2** | **Cells + S1D3** | **Cells + S1D4** | **Cells + S1D5** | **Cells + S1D6** | **Cells + S1D7** | **Cells + S1D8** | **Cells  + negative control** | **Cells  +  positive control** | Cell culture medium |
| **E** | Cell culture medium | **Cells + S2D1** | **Cells + S2D2** | **Cells + S2D3** | **Cells + S2D4** | **Cells + S2D5** | **Cells + S2D6** | **Cells + S2D7** | **Cells + S2D8** | **Cells  + negative control** | **Cells  +  positive control** | Cell culture medium |
| **F** | Cell culture medium | **Cells + S2D1** | **Cells + S2D2** | **Cells + S2D3** | **Cells + S2D4** | **Cells + S2D5** | **Cells + S2D6** | **Cells + S2D7** | **Cells + S2D8** | **Cells  + negative control** | **Cells  +  positive control** | Cell culture medium |
| **G** | Cell culture medium | **Cells + S2D1** | **Cells + S2D2** | **Cells + S2D3** | **Cells + S2D4** | **Cells + S2D5** | **Cells + S2D6** | **Cells + S2D7** | **Cells + S2D8** | **Cells  + negative control** | **Cells  +  positive control** | Cell culture medium |
| **H** | Cell culture medium | **S2D1** | **S2D2** | **S2D3** | **S2D4** | **S2D5** | **S2D6** | **S2D7** | **S2D8** | **Negative  control** | **Positive control** | Cell culture medium |

S: sample, D: dilution; cells are seeded in the central part of the plate (surrounded in black).

### 2.3.2. WST-1 assay

**Fig. S1** WST-1 assay results for NonPEG, P21 and P51 after they were diluted in NaCl 0.9% solution or sucrose 5% solution (N=3, n=3); incubation time: 6h

**Fig. S2** PI assay assay results for NonPEG, P21 and P51 after they were diluted in Nacl 0.9% solution or sucrose 5% solution (N=3, n=3); incubation time: 6h
